# Periostin and TGFβ1 levels in the saliva and gingival fluid of periodontitis patients: a cross-sectional study

**DOI:** 10.1590/1807-3107bor-2026.vol40.048

**Published:** 2026-07-27

**Authors:** Mehmet Akif TURKAN, Mehmet Oguzhan ERGIN, Kamile ERCIYAS

**Affiliations:** (a) Private Dental Practice, Istanbul, Turkey; (b) Harran University, School of Dentistry, Department of Periodontology, Şanlıurfa, Turkey.; (c) Gaziantep University, School of Dentistry, Department of Periodontology, Gaziantep, Turkey.

**Keywords:** Gingival Crevicular Fluid, Periodontitis, Saliva, Periostin, Transforming Growth Factor beta

## Abstract

Periodontitis is a chronic infectious disease characterized by the progressive destruction of tooth-supporting tissues. Identifying reliable, noninvasive biomarkers is essential for improving early detection and monitoring. This cross-sectional case‒control study evaluated clinical periodontal parameters and quantified periostin and transforming growth factor-beta 1 (TGF-β1) levels in the saliva and gingival crevicular fluid (GCF) of patients with stage I–II periodontitis and periodontally healthy controls. A total of 60 systemically healthy, nonsmoking participants aged 20–60 years were included (30 periodontitis patients and 30 periodontally healthy controls). Clinical periodontal parameters, including the plaque index, gingival index, probing depth, and clinical attachment level, were recorded. GCF and saliva samples were analyzed using an enzyme-linked immunosorbent assay (ELISA). Compared with periodontally healthy controls, stage I–II periodontitis patients had significantly greater numbers of clinical periodontal parameters (p < 0.001). They also had elevated salivary periostin (1,234.5 ± 505.82 ng/mL vs. 596.32 ± 320.47 ng/mL; p = 0.001), GCF TGF-β1 (10.14 ± 4.44 pg/mL vs. 6.50 ± 3.13 pg/mL; p = 0.003), and salivary TGF-β1 (31.59 ± 7.69 pg/mL vs. 20.25 ± 9.48 pg/mL; p = 0.001) levels. GCF periostin levels did not significantly differ between the periodontitis group (311.3 ± 210.3 ng/mL) and the periodontally healthy control group (208.6 ± 147.6 ng/mL) (p = 0.092). These findings suggest that salivary periostin and TGF-β1, together with GCF TGF-β1, may serve as promising noninvasive biomarkers for the diagnosis and monitoring of early-stage periodontitis. Salivary periostin showed particularly strong discriminatory potential and may hold promise for future chairside diagnostic applications.

## Introduction

The periodontium comprises the gingiva, alveolar bone, periodontal ligament (PDL), cementum, and surrounding connective tissues, all of which are essential for tooth support and periodontal homeostasis.^1,2^ Inflammatory conditions affecting these structures are broadly classified as gingivitis, a reversible inflammatory process confined to the gingiva, and periodontitis, a chronic, multifactorial disease characterized by a dysbiotic biofilm and a dysregulated host immune–inflammatory response, leading to progressive destruction of the periodontal ligament, connective tissue attachment loss, and alveolar bone resorption.^1,2^ Periodontitis is among the most prevalent chronic diseases worldwide and represents a major public health concern because of its association with tooth loss, impaired oral function, and systemic health implications.^3^

Conventional periodontal diagnosis relies primarily on clinical parameters such as probing pocket depth (PPD), clinical attachment level (CAL), and radiographic bone loss. Although these measures are indispensable for disease classification and staging, they predominantly reflect past tissue destruction and provide limited information regarding current disease activity or future progression.^4^ Although contemporary classification and grading systems aim to improve diagnostic precision, their ability to accurately predict disease dynamics remains restricted. As emphasized by Tonetti et al.,^1^ current periodontal classifications highlight an evident gap regarding the integration of biological markers, underscoring the need for biomarker-based approaches to enhance diagnosis and risk assessment. This diagnostic limitation has intensified research into molecular biomarkers capable of providing real-time insights into periodontal inflammation and tissue remodeling.

In this context, gingival crevicular fluid (GCF) and saliva have gained increasing attention as diagnostic media because of their accessibility, minimal invasiveness, and high abundance of host-derived inflammatory mediators, enzymes, and tissue degradation products.^5,6^ GCF is collected directly from the gingival sulcus and reflects site-specific inflammatory and immune responses, whereas saliva represents a pooled sample derived from multiple periodontal sites and salivary glands, reflecting the overall oral inflammatory burden. Accordingly, while GCF provides localized information, saliva offers a broader overview of periodontal disease status, making their combined evaluation complementary in periodontal diagnostics.^7^

Among the numerous candidate biomarkers investigated in periodontal research, periostin and TGF-β1 have attracted particular interest because of their pivotal roles in periodontal tissue homeostasis and immune regulation. Periostin is a matricellular protein of the fasciclin family that is abundantly expressed in connective tissues such as the periodontal ligament, periosteum, and heart valves.^8,9^ It contributes to extracellular matrix organization, mechanical stability, and the modulation of cell adhesion and differentiation. Experimental studies have demonstrated that periostin deficiency disrupts periodontal ligament integrity and promotes alveolar bone loss, highlighting its importance in maintaining periodontal health.^10^ However, clinical studies evaluating periostin levels in periodontal disease have yielded conflicting results, with both increased and decreased levels reported depending on disease severity, sampling method, and biological medium.^11-13^

Transforming growth factor-beta 1 (TGF-β1) is a multifunctional cytokine involved in immune modulation, extracellular matrix synthesis, and epithelial–mesenchymal interactions.^14,15^ Its biological effects are highly context-dependent and involve both proinflammatory and anti-inflammatory functions. Elevated levels of TGF-β1 in the GCF and saliva of periodontitis patients have been suggested to reflect compensatory mechanisms related to tissue repair and remodeling during chronic inflammation. The potential interaction between periostin and TGF-β1 may represent a coordinated host response to periodontal tissue injury and matrix degradation. Despite their biological relevance, existing studies investigating these biomarkers are limited by heterogeneous sampling protocols, variable disease classifications, and differences in study populations.^16^

Therefore, the aim of the present study was to simultaneously evaluate periostin and TGF-β1 levels in both saliva and gingival crevicular fluid, together with comprehensive clinical periodontal parameters, in systemically healthy participants belonging to the Stage I–II periodontitis group and the periodontally healthy control group.

Periostin was selected because of its role in maintaining periodontal ligament integrity and extracellular matrix organization, whereas TGF-β1 was included because of its involvement in inflammatory regulation and tissue regeneration. The combined assessment of these biomarkers in two complementary biological fluids may provide further insight into host responses in early stage periodontal disease and support the development of noninvasive diagnostic strategies.

Ethical Approval: Gaziantep University Clinical Research Ethics Committee, 06.03.2019 - 2019/66.

## Methods

### Participants and clinical evaluations

This was an observational, cross-sectional, single-center case–control study designed to investigate the diagnostic value of salivary and GCF periostin and TGF-β1 levels in the Stage I–II periodontitis group. The study was conducted at the Department of Periodontology, Faculty of Dentistry, Gaziantep University, between March and June 2019 and was reported in accordance with the Strengthening the Reporting of Observational Studies in Epidemiology (STROBE) guidelines.^17^ Periodontal status was classified according to the 2018 World Workshop criteria, and participants were allocated to either the Stage I–II periodontitis group or the periodontally healthy control group.^1^

All clinical periodontal measurements were performed by a single trained examiner using standardized periodontal assessment protocols to minimize measurement variability.

Formal intraexaminer reproducibility statistics (e.g., kappa or ICC) were not recorded; however, the use of a single experienced examiner and standardized procedures was intended to reduce variability.

The examiner was blinded to the biochemical analysis results, and all laboratory analyses were conducted by an investigator who was blinded to the clinical status of the participants; owing to the nature of the clinical examination, blinding to periodontal status was not feasible.

Clinical periodontal parameters included the plaque index (PI), gingival index (GI), probing pocket depth (PPD), and clinical attachment level (CAL). GI was assessed according to the Löe and Silness criteria based on visual evaluation of gingival color, edema, and inflammatory changes, without the use of bleeding on probing as a scoring criterion.^18^ Measurements were performed using a Williams periodontal probe (Hu-Friedy, Chicago, USA). PI and GI were recorded on four surfaces per tooth (mesiobuccal, mid-buccal, distobuccal, and mid-palatal/lingual), whereas PPD and CAL were measured at six sites per tooth (mesiobuccal, mid-buccal, distobuccal, mesiolingual/palatal, mid-lingual/palatal, and distolingual/palatal).

The sample size calculation was performed before data collection using GPower version 3.1 (Heinrich Heine University, Düsseldorf, Germany) for an independent samples t test (one-tailed, effect size = 0.80, α = 0.05, statistical power = 0.80), indicating that a minimum of 42 participants (21 per group) was needed.^19^

Potential confounding was minimized by recruiting nonsmoking, systemically healthy participants, restricting periodontitis cases to Stages I–II, and aiming for a balanced sex distribution across groups; no multivariate statistical adjustment was performed.

Ethical approval was obtained from the Clinical Research Ethics Committee of Gaziantep University (decision number 2019/66, dated 06.03.2019). Written informed consent was obtained from all participants in compliance with the Declaration of Helsinki.^20^

**Figure 1:**
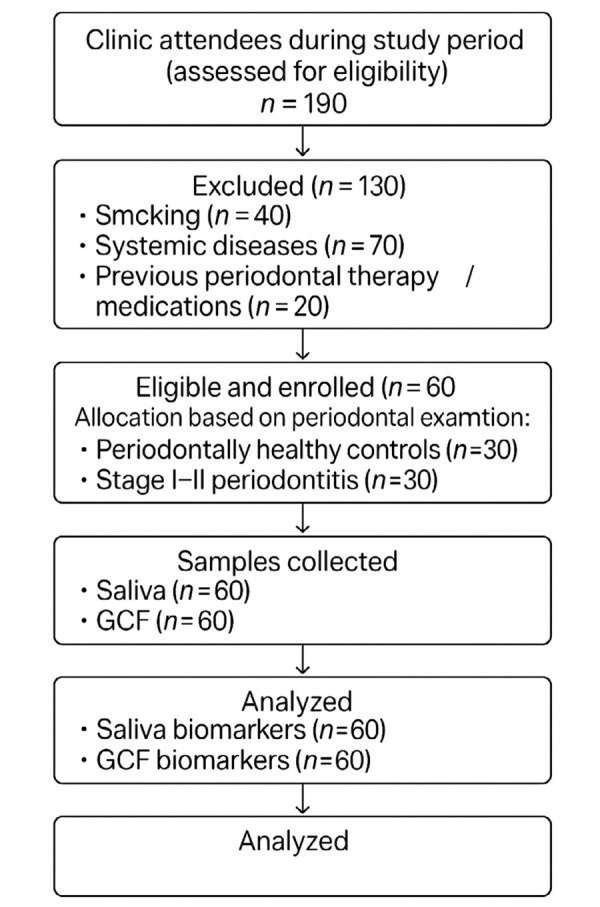
Flow diagram of participant recruitment, exclusion, and final allocation to the periodontitis and periodontally healthy control groups.

The patient selection process, including the numbers of screened, excluded, and finally included participants, is illustrated in Figure 1.

The flowchart illustrates the participant selection process based on the predefined inclusion and exclusion criteria. Individuals exhibiting no systemic health issues were screened and allocated into two groups: those exhibiting periodontal health and those diagnosed with Stage I-II periodontitis. Participants who met all the study’s criteria and were deemed eligible were included in the final statistical analyses.

### Inclusion and exclusion criteria

#### Inclusion criteria

1. Age range and general characteristics: participants were recruited within a predefined age range of 20–60 years, and age distributions between groups were kept as comparable as possible to minimize confounding. An age range of 20–60 years was chosen to ensure the inclusion of both younger and middle-aged adults, thereby providing a representative sample of the adult population commonly affected by periodontitis. Restricting the study to this interval minimized the confounding effect of adolescent periodontal changes and excluded older individuals in whom age-related systemic conditions and tooth loss could bias biomarker levels. Thus, this broad but controlled range allowed the data to be generalized across a wider spectrum of adults while maintaining internal validity.
2. Systemic health status: systemic health status was confirmed through a detailed medical history and cross-checked against institutional clinical records.
3. Periodontally healthy control group: periodontally healthy control participants were defined according to the 2018 World Workshop classification and presented no interdental clinical attachment loss, buccal/oral clinical attachment loss ≤3 mm, probing pocket depths within physiological limits, and no radiographic evidence of periodontal bone loss.^1^
4. Periodontitis group: the Stage I–II periodontitis group consisted of nonsmoking individuals without diabetes mellitus, diagnosed according to the 2018 World Workshop classification, and characterized by interdental clinical attachment loss at ≥2 nonadjacent teeth, probing pocket depths ≤5 mm, and radiographic findings consistent with early stage periodontal bone loss.^1^

#### Exclusion criteria

1. Tooth loss related to periodontitis: participants with a history of tooth loss due to periodontitis were excluded based on clinical examination, radiographic evaluation, and dental history. The total number of missing teeth, regardless of cause, was not recorded and therefore was not included in the statistical analysis.
2. Radiographic considerations: radiographic examinations were performed for diagnostic confirmation and to exclude participants with periodontitis-related tooth loss; however, quantitative radiographic measurements or bone loss indices were not recorded or statistically analyzed. Diagnosis and staging were based on the 2018 World Workshop criteria using clinical parameters (PPD and CAL) and qualitative radiographic assessment.
3. Medical and behavioral exclusion criteria: participants with diabetes mellitus, cardiovascular disease, immunological disorders, or any condition requiring regular medication were excluded. Pregnant or lactating women, smokers, and individuals who had received periodontal therapy within the previous 6 months, antibiotics within the past 6 months, or anti-inflammatory medications within the past 3 months were also excluded.

## Collection of GCF and saliva samples

GCF samples were collected 24 hours after clinical measurements from two sites per jaw quadrant with the deepest probing depths. Sterilized paper strips (Periopaper®, OraFlow Inc., Plainview, USA) were gently inserted into the gingival sulcus until slight resistance was felt (Brill Technique)^21^ and left for 30 seconds. Sites were isolated with cotton tampons, plaque was removed, and areas were dried with an air spray to prevent saliva or blood contamination. Contaminated samples were excluded. GCF volume was measured using a precalibrated Periotron 8000 and converted to microliters via Periotron software (OraFlow Inc.).

Unstimulated saliva samples (5 mL) were collected between 09:00 and 11:00, following a two-hour fasting period, using a modified drainage method.^22^ The samples were subsequently centrifuged at 1000 rpm for 10 minutes, after which the supernatant was transferred to Eppendorf tubes (0.5 mL per tube), which were subsequently sealed with paraffin wax and stored at -80°C until analysis.

## Measurements of GCF and Saliva Periostin and TGF-β1 Levels

Periostin and TGF-β1 levels were quantified using ScienCell-BosterBio ELISA kits (Human TGF-β1 PicoKine^TM^ and Human Periostin/OSF2 PicoKineTM) based on the sandwich ELISA principle. The samples and reagents were brought to room temperature, and the absorbance was measured at 450 nm using an ELISA reader (Biotek, ELx800, USA).

## Statistical analysis

The normality of the data distribution was assessed using the Shapiro–Wilk test. For comparisons between two independent groups, variables with a normal distribution were analyzed using the independent-samples Student’s t test, whereas variables without a normal distribution were analyzed using the Mann–Whitney U test. Categorical variables were compared using the chi-square test, and correlations between numerical variables were evaluated using Spearman’s rank correlation coefficient. For all key continuous variables, 95% confidence intervals (CIs) were calculated. All statistical analyses were performed using SPSS for Windows, version 22.0 (IBM Corp., Armonk, USA), and a p value of < 0.05 was considered to indicate statistical significance. All statistical comparisons were performed between the Stage I–II periodontitis group and the periodontally healthy control group.

A priori sample size calculations were conducted for comparisons between these two independent groups (with and without periodontitis).

Demographic variables, including age and sex distribution, clinical periodontal parameters, and biomarker levels (periostin and TGF-β1 in saliva and GCF) were compared between the two groups using appropriate statistical tests based on data distribution. Receiver operating characteristic (ROC) curve analyses were performed to evaluate the diagnostic potential of salivary and GCF periostin and TGF-β1 levels in distinguishing patients with Stage I–II periodontitis from periodontally healthy controls. The area under the curve (AUC), optimal cutoff value, sensitivity, and specificity were calculated.

## Results

### Demographic characteristics and clinical parameters

Participants were consecutively recruited between March and June 2019 from patients attending the Department of Periodontology, Faculty of Dentistry, Gaziantep University, for routine periodontal examination or treatment.

The study included 60 systemically healthy individuals aged between 20 and 60 years. The mean age was 41.27 ± 4.7 years in the Stage I–II periodontitis group and 37.33 ± 5.45 years in the periodontally healthy control group. The difference in mean age between the two groups was statistically significant (p = 0.004). Each group consisted of 15 males and 15 females, with no significant difference in sex distribution (p = 1.000). (Tables 1 and 2).

**Table 1:** Gender distribution of the study groups.

Gender	Periodontally healthy (n = 30)		Stage I–II periodontitis (n = 30)		p-value
	n	% (95%CI)	n	% (95%CI)	
Male	15	50.0 (32.1–67.9)	15	50.0 (32.1–67.9)	1000
Female	15	50.0 (32.1–67.9)	15	50.0 (32.1–67.9)	

Data are presented as numbers (N) and percentages (%). Group comparisons were performed using the chi-square test.

**Table 2:** Age distribution of the study groups.

Variable	Periodontally Healthy (n = 30)	Median (25%–75%)	Stage I–II Periodontitis (n = 30)	Median (25%–75%)	p-value
	Mean ± SD (95%CI)		Mean ± SD (95% CI)		
Age (years)	37.33 ± 5.45 (35.29–39.37)	37 (34–41)	41.27 ± 4.70 (39.51–43.03)	42.5 (39–45)	0.004

Data are presented as the mean ± standard deviation (SD), median (25th–75th percentile), and 95% confidence interval (CI). Group comparisons were performed using Student’s t test or the Mann–Whitney U test, as appropriate.

Clinical periodontal parameters, including the plaque index (PI), gingival index (GI), probing pocket depth (PPD), and clinical attachment level (CAL), were significantly greater in the Stage I–II periodontitis group than in the periodontally healthy control group (p < 0.001 for all comparisons). The GCF volume was also significantly greater in the Stage I–II periodontitis group (0.89 ± 0.21 µL vs. 0.27 ± 0.14 µL, p < 0.001). In accordance with the eligibility criteria, no participants presented with tooth loss related to periodontitis, and the radiographic findings supported the group classification (Stage I–II periodontitis versus no alveolar bone loss). The detailed data are presented in Table 3.

**Table 3:** Clinical parameters.

Parameter	Periodontally Healthy (n = 30)	Median (25%–75%)	Stage I–II Periodontitis (n = 30)	Median (25%–75%)	p-value
	Mean ± SD (95%CI)		Mean ± SD (95%CI)		
PI	1.40 ± 0.22 (1.318–1.482)	1.33 (1.21–1.56)	2.26 ± 0.12 (2.215–2.305)	2.29 (2.16–2.35)	0.001
GI	1.45 ± 0.21 (1.372–1.528)	1.45 (1.29–1.60)	2.25 ± 0.10 (2.213–2.287)	2.25 (2.20–2.31)	0.001
PPD (mm)	1.85 ± 0.17 (1.787–1.913)	1.86 (1.73–1.97)	3.99 ± 0.28 (3.885–4.095)	3.99 (3.91–4.13)	0.001
CAL (mm)	0.00 ± 0.00 (0.000–0.000)	0 (0–0)	3.99 ± 0.28 (3.885–4.095)	3.99 (3.91–4.13)	0.001

Data are presented as the mean ± standard deviation (SD), median (25th–75th percentile), and 95% confidence interval (CI). Variables with a normal distribution were analyzed using Student’s t test, whereas variables without a normal distribution were analyzed using the Mann–Whitney U test. PI: Plaque index; GI: Gingival index; PPD: Probing pocket depth; CAL: Clinical attachment level. p < 0.05 indicates statistical significance.

### Periostin and TGF-β1 levels in GCF and saliva

Salivary periostin levels were significantly higher in the Stage I–II periodontitis group (1,234.5 ± 505.82 ng/mL) than in the periodontally healthy control group (596.32 ± 320.47 ng/mL) (p = 0.001). GCF periostin levels were also elevated in the Stage I–II periodontitis group (311.3 ± 210.3 ng/mL) compared with those in the periodontally healthy control group (208.62 ± 147.61 ng/mL), although this difference did not reach statistical significance (p = 0.092). TGF-β1 levels were significantly elevated in both saliva and GCF samples from the Stage I–II periodontitis group. Salivary TGF-β1 concentration was 31.59 ± 7.69 pg/mL in the periodontitis group and 20.25 ± 9.48 pg/mL in the periodontally healthy control group (p = 0.001). The concentration of GCF-derived TGF-β1 was 10.14 ± 4.44 pg/mL and 6.50 ± 3.13 pg/mL, respectively (p = 0.003). These results are summarized in Table 4.

**Table 4:** Comparison of biochemical parameters and gingival crevicular fluid (GCF) volume between the study groups.

Parameter	Periodontally Healthy (n = 30)	Median (25%–75%)	Stage I-II Periodontitis (n = 30)	Median (25%–75%)	p-value
	Mean ± SD (95% CI)		Mean ± SD (95% CI)		
GCF-PERIOSTİN (ng/mL)	208.62 ± 147.61 (153.50–263.74)	160.48 (102.09–237.50)	311.30 ± 210.30 (232.77–389.83)	272.83 (115.83–473.33)	0.092
Salivary PERIOSTİN (ng/mL)	596.32 ± 320.47 (476.65–715.99)	569.78 (379.96–831.91)	1234.50 ± 505.82 (1045.62–1423.38)	1286.06 (842.72–1538.59)	0.001
GCF-TGFβ1 (pg/mL)	6.50 ± 3.13 (5.33–7.67)	6.49 (4.21–7.76)	10.14 ± 4.44 (8.48–11.80)	9.45 (5.91–14.20)	0.003
Salivary TGFβ1 (pg/mL)	20.25 ± 9.48 (16.71–23.79)	23.20 (10.26–26.56)	31.59 ± 7.69 (28.72–34.46)	32.74 (26.83–37.34)	0.001
GCF volume (µL)	0.27 ± 0.14 (0.218–0.322)	0.26 (0.16–0.40)	0.89 ± 0.21 (0.812–0.968)	0.90 (0.67–1.11)	0.001

Data are presented as the mean ± standard deviation (SD), median (25th–75th percentile), and 95% confidence interval (CI). Variables with a normal distribution were analyzed using Student’s t test, whereas variables without a normal distribution were analyzed using the Mann–Whitney U test. GCF: Gingival crevicular fluid; TGF-β1: Transforming growth factor beta-1. p < 0.05 indicates statistical significance.

### Correlations between clinical parameters and biomarker levels

In the Stage I–II periodontitis group, GCF periostin levels were significantly positively correlated with GCF TGF-β1 concentrations (r = 0.497, p = 0.007) (Table 5).

**Table 5:** Within-group correlations between salivary and gingival crevicular fluid (GCF) periostin and TGF-β1 levels.

Variables	GCF Periostin (ng/mL)	Salivary Periostin (ng/mL)	GCF TGF-β1 (pg/mL)	Salivary TGF-β1(pg/mL)
Stage I–II Periodontitis group				
GCF Periostin	—	r = 0.130, p = 0.503	r = 0.128, p = 0.533	r = –0.422*, p = 0.025
Salivary Periostin		—	r = –0.210, p = 0.265	r = 0.217, p = 0.250
GCF TGF-β1			—	r = 0.497**, p = 0.007
Salivary TGF-β1				—
Periodontally healthy controls				
GCF Periostin	—	r = –0.244, p = 0.229	r = 0.140, p = 0.469	
Salivary Periostin		—	r = –0.187, p = 0.341	r = 0.037, p = 0.848
GCF TGF-β1			—	r = –0.062, p = 0.767
Salivary TGF-β1				—

In the periodontally healthy control group, the mean full-mouth plaque index was moderately and significantly negatively correlated with GCF TGF-β1 levels (r = –0.405, p = 0.040), whereas the mean full-mouth gingival index demonstrated a statistically significant weak negative correlation with GCF periostin levels (r = –0.419, p = 0.024) (Table 6).

**Table 6:** Values are Spearman’s rank correlation coefficients (r) with corresponding p values. *p < 0.05 indicates statistical significance; *p < 0.01 indicates high statistical significance. GCF: Gingival crevicular fluid; TGF-β1: Transforming growth factor beta-1. . Correlations of laboratory findings with clinical parameters in the study groups.

Variables	GCF–Periostin (ng/mL)	Saliva–Periostin (ng/mL)	GCF–TGFβ1 (pg/mL)	Saliva–TGFβ1 (pg/mL)	GCF–Periostin (ng/mL)	Saliva–Periostin (ng/mL)	GCF–TGFβ1 (pg/mL)	Saliva–TGFβ1 (pg/mL)
n	29	30	26	29	30	30	28	30
Age (years) - r	-0.045	0.316	-0.041	0.080	0.114	-0.242	0.195	0.337
p-value	0.818	0.089	0.841	0.681	0.548	0.198	0.320	0.068
PI - r	-0.279	0.272	-0.405*	-0.018	0.303	-0.222	0.024	0.033
p-value	0.143	0.146	0.040	0.925	0.104	0.239	0.905	0.864
GI - r	-0.419*	0.107	-0.169	-0.107	0.065	-0.048	0.116	-0.092
p-value	0.024	0.575	0.410	0.580	0.734	0.802	0.556	0.629
PPD - r	0.240	0.067	0.128	-0.240	0.103	-0.184	0.366	-0.224
p-value	0.210	0.723	0.534	0.210	0.589	0.331	0.055	0.234
CAL - r					0.103	-0.184	0.366	-0.224
p-value					0.589	0.331	0.055	0.234

The values are Spearman’s correlation coefficients (r) with corresponding p values. p < 0.05 indicates statistical significance; p < 0.01 indicates high statistical significance.

### ROC curve analysis

**Figure 2:**
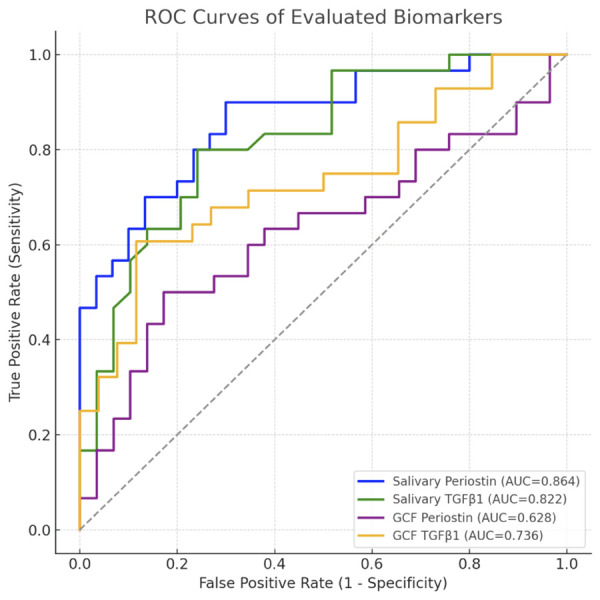
Receiver operating characteristic (ROC) curves showing the diagnostic performance of salivary periostin, salivary TGF-β1, gingival crevicular fluid (GCF) periostin, and GCF TGF-β1 in distinguishing patients with stage I–II periodontitis from periodontally healthy controls.

ROC curve analyses were conducted to evaluate the diagnostic performance of the studied biomarkers. Salivary periostin showed good accuracy in distinguishing the Stage I–II periodontitis group from the periodontally healthy control group, with an AUC of 0.864 (95%CI: 0.774–0.955; p < 0.001). At an optimal cutoff value of approximately 711.5 ng/mL, the sensitivity and specificity were 90% and 70%, respectively. Salivary TGF-β1 also exhibited good diagnostic ability, with an AUC of 0.822 (95%CI: 0.716–0.929; p < 0.001); at a cutoff of 26.7 pg/mL, the sensitivity and specificity were 80% and 75.9%, respectively. In contrast, GCF periostin showed limited diagnostic utility, with an AUC of 0.628 (95%CI: 0.481–0.774; p = 0.089), a sensitivity of 50%, and a specificity of 82.8% at the optimal cutoff (317.9 ng/mL). The diagnostic accuracy of GCF TGF-β1 was moderate, with an AUC of 0.736 (95% CI: 0.601–0.872; p < 0.001), and 60.7% sensitivity and 88.5% specificity were achieved at a cutoff of 8.85 pg/mL. The detailed results are presented in Table 7 and Figure 2.

**Table 7:** ROC Curve Analysis of Salivary and GCF Biomarkers.

Biomarker	AUC (95%CI)	p-value	Cutoff	Sensitivity 9%)	Specificity (%)
Salivary periostin	0.864 (0.774–0.955)	< 0.001	711.5	90	70
Salivary TGF-β1	0.822 (0.716–0.929)	< 0.001	26.7	80	75.9
GCF periostin	0.628 (0.481–0.774)	0.089	317.9	50	82.8
GCF TGF-β1	0.736 (0.601–0.872)	< 0.001	8.85	60.7	88.5

AUC: area under the curve; CI: confidence interval; ROC: receiver operating characteristic; GCF: gingival crevicular fluid; TGF-β1: transforming growth factor beta-1.

The receiver operating characteristic (ROC) curve analysis of salivary and gingival crevicular fluid (GCF), periostin and transforming growth factor-beta 1 (TGF-β1) was performed to discriminate Stage I–II periodontitis from periodontally healthy controls. The area under the curve (AUC) is a metric used to quantify the overall diagnostic accuracy of a test. It is calculated as the true-positive rate (sensitivity) minus the false-positive rate (1 - specificity). The optimal cut-off values, sensitivity, and specificity were derived from the ROC analysis.

## Discussion

In the present study, all clinical periodontal parameters and gingival crevicular fluid volume were significantly greater in the Stage I–II periodontitis group, confirming appropriate disease classification and consistency with previous reports.^11,12^ This robust clinical characterization provides a reliable framework for the interpretation of biomarker findings. Within this context, it is noteworthy that no previous study has simultaneously quantified periostin and TGF-β1 levels in both saliva and gingival crevicular fluid in patients with early stage (Stage I–II) periodontitis to evaluate their combined diagnostic potential. Previous studies have focused primarily on advanced forms of periodontitis, reporting reduced periostin levels in gingival crevicular fluid, with progressively lower concentrations observed as disease severity increases.^11,12^ By contrast, no statistically significant difference in GCF periostin levels was detected between the groups in the present study, which focused exclusively on stage I–II periodontitis. This discrepancy may reflect the dynamic balance between extracellular matrix degradation and reparative processes during early-stage periodontal inflammation. In such stages, periostin expression may be maintained as part of an adaptive host response aimed at preserving periodontal ligament integrity rather than reflecting irreversible tissue breakdown.^23,24^Differences in disease severity, sampling methods, normalization to GCF volume, and analytical methods may further contribute to variability among studies.

Salivary periostin levels were significantly higher in the Stage I–II periodontitis group than in the periodontally healthy control group, in agreement with the findings of Aral et al.^12^ Because saliva integrates inflammatory and tissue remodeling signals from multiple periodontal sites, elevated salivary periostin levels may reflect cumulative periodontal tissue responses rather than site-specific changes. This characteristic may explain the greater sensitivity of salivary periostin in detecting early stage periodontal alterations than that of GCF measurements and supports its potential role as a supportive noninvasive biomarker.

ROC curve analyses further highlighted the diagnostic potential of salivary periostin and TGF-β1 in distinguishing stage I–II periodontitis from periodontal health. The superior discriminatory performance of salivary biomarkers may be attributed to the ability of saliva to reflect the overall oral inflammatory burden, thereby enhancing diagnostic accuracy in early disease stages. By contrast, the limited diagnostic performance of GCF periostin is consistent with the absence of statistically significant intergroup differences observed in the present study.

TGF-β1 levels were significantly elevated in both the GCF and saliva samples of the Stage I–II periodontitis group, supporting the findings of previous reports linking this cytokine to periodontal inflammation and tissue remodeling.^14,15^ Elevated TGF-β1 expression may represent compensatory regulatory mechanisms aimed at modulating chronic inflammation and promoting extracellular matrix repair. Gürkan et al. reported positive associations between GCF TGF-β1 levels and clinical periodontal parameters, whereas Atilla et al. indicated that genetic variations in TGF-β1 may influence individual susceptibility to periodontitis.^14,25^ Collectively, these findings reinforce the biological relevance of TGF-β1 in the pathophysiology of periodontal disease.

Several limitations should be acknowledged. The cross-sectional design precludes causal inference and restricts conclusions regarding disease progression.

In addition, the mean age of the periodontitis group was significantly greater than that of the periodontally healthy control group, and no statistical adjustment for age was performed. Age-related alterations in connective tissue metabolism and immune regulation may partially influence periostin and TGF-β1 expression. Periostin is closely associated with fibroblast activity, extracellular matrix turnover, and periodontal ligament integrity, whereas TGF-β1 plays a central role in the age-dependent modulation of inflammatory responses and tissue remodeling.^11,14,23^ Therefore, age-related biological differences may have contributed, at least in part, to the observed biomarker profiles and should be considered when the findings are interpreted.

Moreover, although radiographic examinations were used for diagnostic confirmation, quantitative radiographic measurements were not recorded or statistically analyzed, which may have reduced the precision of disease staging. While tooth loss due to periodontitis was applied as an exclusion criterion, the total number of missing teeth, regardless of cause, was not recorded, precluding the evaluation of cumulative periodontal destruction. Finally, the absence of multivariate statistical analyses to control for potential confounding factors highlights the need for future longitudinal studies incorporating age-matched populations, standardized radiographic assessments, and comprehensive statistical adjustment.

## Conclusion

Within the limitations of this cross-sectional study, salivary periostin and TGF-β1 levels were found to be higher in individuals with Stage I–II periodontitis than in periodontally healthy controls. These findings suggest that salivary biomarkers may reflect early changes in periodontal inflammation. However, owing to the cross-sectional design and the absence of longitudinal follow-up, no causal or predictive inferences can be drawn. Further well-designed longitudinal studies with age-matched populations and comprehensive statistical adjustments are needed to clarify the clinical relevance and potential diagnostic value of these biomarkers in early stage periodontitis.

## Data Availability

The authors declare that all data generated or analyzed during this study are included in this published article.
